# Nutritional management recommendation systems in polycystic ovary syndrome: a systematic review

**DOI:** 10.1186/s12905-024-03074-3

**Published:** 2024-04-12

**Authors:** Leila Shahmoradi, Leila Azadbakht, Jebraeil Farzi, Sharareh Rostam Niakan Kalhori, Alireza Banaye Yazdipour, Fahimeh Solat

**Affiliations:** 1 Health Information Management and Medical Informatics Department, School of Allied Medical Sciences, Tehran University of Medical Sciences, Tehran, Iran; 2 Department of Community Nutrition, School of Nutritional Sciences and Dietetics, Tehran University of Medical Sciences, Tehran, Iran; 3 Endocrinology and Metabolism Research Centre, Tehran University of Medical Sciences, Tehran, Iran; 4 Health Information Technology Department, School of Allied Medical Sciences, Zabol University of Medical Sciences, Zabol, Balouchestan, Sistan Iran; 5 Students’ Scientific Research Center (SSRC), Tehran University of Medical Sciences, Tehran, Iran; 6 Department of Health Information Technology, School of Paramedical and Rehabilitation Sciences, Mashhad University of Medical Sciences, Mashhad, Iran; 7 Student Research Committee, Saveh University of Medical Sciences, Saveh, Iran

**Keywords:** Polycystic ovary syndrome, Artificial intelligence, Application, Decision support system, Nutrition recommender system

## Abstract

**Background:**

People with polycystic ovary syndrome suffer from many symptoms and are at risk of developing diseases such as hypertension and diabetes in the future. Therefore, the importance of self-care doubles. It is mainly to modify the lifestyle, especially following the principles of healthy eating. The purpose of this study is to review artificial intelligence-based systems for providing management recommendations, especially food recommendations.

**Materials and methods:**

This study started by searching three databases: PubMed, Scopus, and Web of Science, from inception until 6 June 2023. The result was the retrieval of 15,064 articles. First, we removed duplicate studies. After the title and abstract screening, 119 articles remained. Finally, after reviewing the full text of the articles and considering the inclusion and exclusion criteria, 20 studies were selected for the study. To assess the quality of articles, we used criteria proposed by Malhotra, Wen, and Kitchenham. Out of the total number of included studies, seventeen studies were high quality, while three studies were moderate quality.

**Results:**

Most studies were conducted in India in 2021. Out of all the studies, diagnostic recommendation systems were the most frequently researched, accounting for 86% of the total. Precision, sensitivity, specificity, and accuracy were more common than other performance metrics. The most significant challenge or limitation encountered in these studies was the small sample size.

**Conclusion:**

Recommender systems based on artificial intelligence can help in fields such as prediction, diagnosis, and management of polycystic ovary syndrome. Therefore, since there are no nutritional recommendation systems for these patients in Iran, this study can serve as a starting point for such research.

**Supplementary Information:**

The online version contains supplementary material available at 10.1186/s12905-024-03074-3.

## Introduction

Polycystic ovary syndrome affects 8 to 13% of women of reproductive age worldwide, making it the most common endocrine problem. This condition can cause menstrual disorders, lack of ovulation, obesity, acne, hirsutism, hair loss, and baldness. Long-term complications include endometrial cancer, infertility, insulin resistance, type 2 diabetes, high blood pressure, heart disease, depression, and stress. Short-term consequences and complications may also arise [1]. Early diagnosis of the disease is very important and can reduce the duration of the disease and the mortality rate. Studies show that in many diseases, early diagnosis is difficult for health care providers. On the other hand, patients do not have the information related to self-management and do not have the necessary knowledge to obtain this information. Social withdrawal may become more prevalent as physical symptoms like acne, hair loss, and depression manifest [2–4]. Following diagnosis, implementing lifestyle modifications to address symptoms like elevated cholesterol and insulin resistance is recognized as an innovative therapeutic approach. Given the prevalent overweight status among women with this syndrome, the significance of adhering to a nutritious diet and engaging in physical activity has been underscored. Consequently, the imperative of devising strategies to promote adherence to healthy dietary habits and facilitate weight loss among affected individuals is deemed essential and inevitable [1].

The use of information technology greatly facilitates the prevention, diagnosis and treatment of chronic diseases and increases their accuracy. Among these technologies, it can be mentioned decision support systems, mobile-based applications, virtual reality, augmented reality, and intelligent decision-making systems. These technologies largely solve the challenge of accessing data and evidence-based information for both patients and medical providers [5–10].

In the healthcare domain, recommender systems represent a practical technology enabling self-care through tailored recommendations. Ultrasound imaging can support early disease detection, alleviating healthcare provider workload and expediting diagnosis. This strategy not only conserves resources and reduces expenses but also leverages mobile phone platforms to enhance awareness, disseminate evidence-based knowledge, and foster beneficial shifts in individual behaviors and habits. By harnessing artificial intelligence, a recommender system can enhance dietary practices among patients with PCOS, leveraging the widespread adoption of smartphones to improve access to medical professionals and reliable information [2, 4].

The study conducted by Jan et al. in India in 2022 analyzed six AI models for diagnosing PCOS. They compared these models based on the number of ultrasound images, segmentation, and classification methods. The evaluation highlighted that the Bayesian classifier achieved the highest accuracy of 93.93%. This study underscores the significant potential of AI in diagnosing PCOS and recommends further research to implement this technology effectively [11].

Boyle et al. conducted a study in Australia in 2018 to assess the need for assistance among individuals with PCOS and evaluate mobile health applications in this area. The results show that 98% of participants owned smartphones, 72% had previously used an application for self-care, and 91% expressed willingness to use a PCOS-specific app if available for managing this syndrome. Accurate, evidence-based information was deemed essential in this study, and all the assessed applications met the required quality standards [12].

The research conducted by Portugal et al. in Canada in 2017 focused on the use of machine learning techniques in recommender systems. The study aimed to identify associated issues and assist researchers in implementing these systems more effectively. The findings of the study highlighted various machine learning techniques and their applications, as well as primary and alternative performance criteria [13].

In 2019, Abhari et al. conducted a study in Iran to assess the characteristics of nutritional recommender systems. The study revealed that if these systems are properly designed, implemented, and evaluated, they can serve as effective tools to improve nutrition and promote a healthy lifestyle [14].

The study conducted in India in 2021 by Kaur and colleagues aimed to develop a method for classifying food images to track patients’ meals and provide guidance to nutritionists on recommended tactics and image classification. The research focused on utilizing deep learning approaches, particularly convolutional neural networks (CNNs), to classify Indian food images accurately [15].

After conducting research, we found no systematic review that assesses nutritional recommendations for individuals with PCOS. Given the significance of utilizing companion health and AI in managing chronic conditions like PCOS, this study aims to explore the effects and uses of AI-based systems for PCOS.

### Motivation

The advancement of AI in healthcare has made it necessary to use AI-powered recommender systems for predicting, diagnosing, treating, and managing chronic ailments like PCOS. There are several reasons why this is important. Ultrasound images are necessary to diagnose PCOS. However, inaccuracies in counting follicles, high diagnostic test costs in developing countries, time-consuming tests, doctor workload, and diagnostic errors can lead to inaccurate diagnoses. AI techniques can automatically diagnose diseases using ultrasound images, overcoming challenges [16–18]. Additionally, since this disease is intricate and has no definitive treatment, the current approach involves a combination of medication and lifestyle changes for disease management. Hence, utilizing recommender systems or self-care systems that prioritize healthy nutrition could be beneficial in mitigating symptoms and lessening the likelihood of associated mental health issues [19, 20]. Besides disease prediction and probability estimation, recommender systems can detect suspicious cases based on AI and take action to prevent disease occurrence or early detection [21].

### Contribution

The article aims to explore the use of artificial recommender systems in polycystic ovary syndrome research. We also examine the challenges and limitations of using these systems and algorithms. This article attracts researchers to conduct studies in the field of recommender systems. The sections of this article are as follows:

- Review of studies from the perspective of publication.
- Review studies of characteristics.
- Examining the limitations and challenges of designing recommender systems.

## Materials and methods

### Study design

The current systematic study was designed and implemented based on the Preferred Reporting Items for Systematic Reviews and Meta-Analyses (PRISMA) statement.

### Data sources

In this study, we were six researchers. The first one determined the search strategy and performed the search in PubMed, Scopus, and Web of Science databases from inception until 6 June 2023. To conduct a search for relevant articles, we utilized a combination of keywords from the article abstracts, including “polycystic ovary syndrome”, “recommender system”, “application program”, “artificial intelligence” and “nutritional program”. We also incorporated Medical Subject Headings (Mesh) into our search strategy. For the complete search strategy, please refer to the supplementary file provided (Table S1-S3).

### Selection criteria

One of the researchers determined the inclusion and exclusion criteria based on similar studies, and finally criteria were approved by the supervisor’s opinion. Inclusion and exclusion criteria are as follows:

*Inclusion criteria*:

1. Studies about recommender systems
2. Articles related to PCOS
3. Articled related to diet management in PCOS
4. Studies that developed an AI system or application

*Exclusion criteria*:

1. Review articles, meta-analysis, conference abstracts, letters to the editor, book chapter.
2. Articles that are unrelated to the goals of the current research.
3. Articles whose full text is written in non-English language.
4. Articles whose full text is not available for data extraction.

### Study selection

In this stage, one of the researchers entered all the articles retrieved from the three databases (PubMed, Web of Science, and Scopus) into the Endnote X9 (Thomson Reuters, Toronto, Ontario, Canada) software. Another researcher removed duplicates. Two of researchers separately, checked the title and abstract of the articles. The items that did not match the inclusion and exclusion criteria were excluded from the study. Finally, by studying and examining the full text of the remaining articles, he selected the articles related to the topic as the final articles. In cases where the two researchers had differing opinions, the supervisor provided the final decision.

### Data extraction

A researcher created an Excel form with the guidance of our supervisor. The research team reviewed articles and extracted necessary data elements. The Excel form included data such as the number of articles, publication year and country.

### Quality assessment

Two independent reviewers assessed the quality of studies using the Newcastle-Ottawa quality assessment criteria proposed by Malhotra [17], Wen et al. [16], and Kitchenham et al. [18]. The quality assessment criteria consist of eleven questions: Q1) Are the aims of the study clearly defined?; Q2) Are all study questions answered?; Q3) Are the variables used in the study clearly stated?; Q4) Are AI techniques, such as machine learning, clearly defined?; Q5) Is the data set size appropriate?; Q6) Is the data collection method clearly stated?; Q7) Is the study methodology repeatable?; Q8) Are the results and findings clearly presented?; Q9) Are the performance measures used to assess the model(s) clearly stated?; Q10) Are the limitations of the study stated?; Q11) Does the research have value for the academic or industry community? The questions were ranked based on three values: “Yes = 2”, “Partial = 1”, or “No = 0”. Each study could obtain a maximum score of 22 and a minimum score of 0. Criteria used to rank the quality assessment of each study include: i) ≤ 49% = Low quality; ii) 50% and 69% = Moderate quality; iii) above 70% = High quality.

## Results

### Study selection

**Fig. 1:**
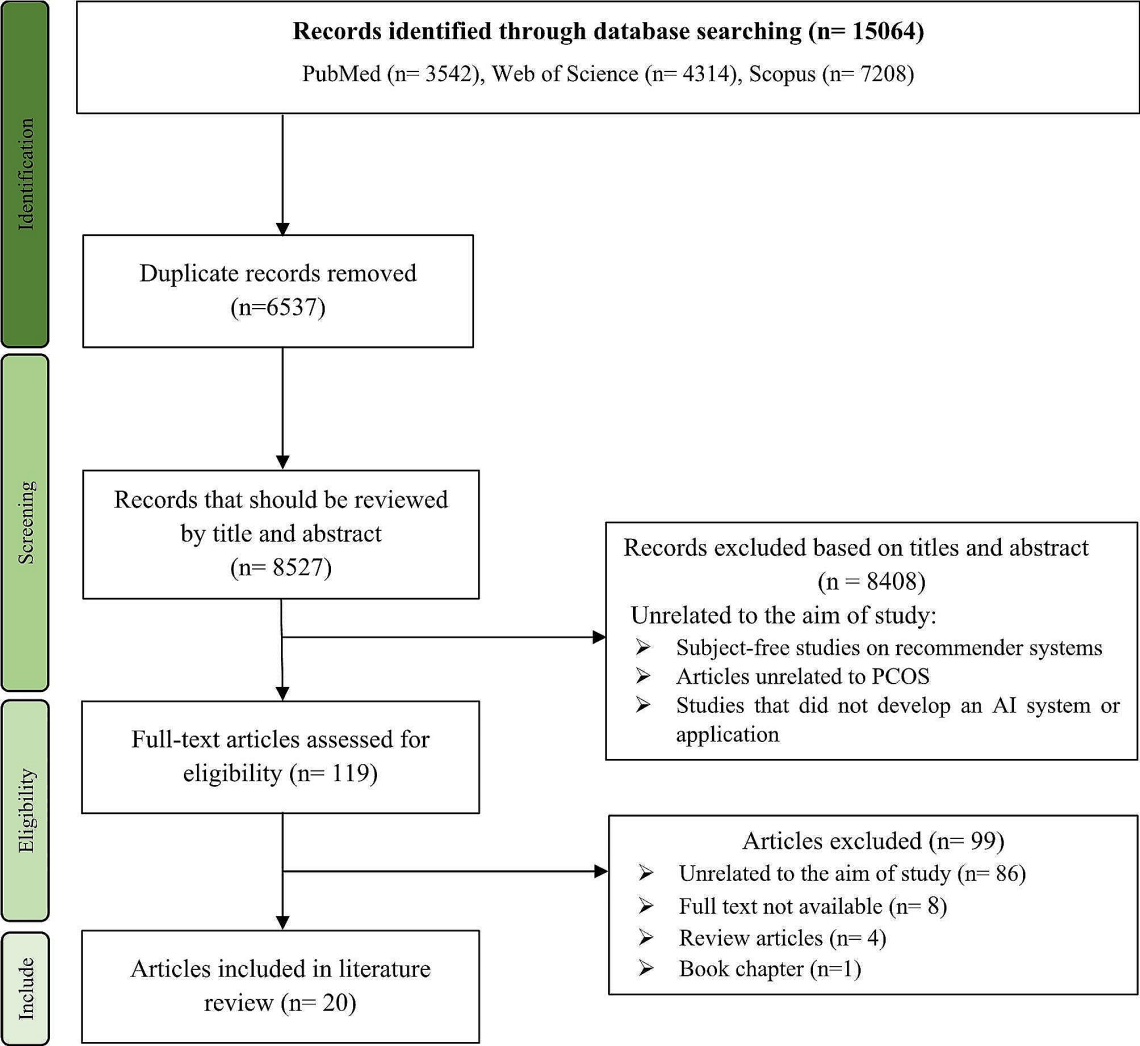
PRISMA flow diagram indicating results of identification and screening process for included and excluded papers

Figure 1 shows the process of searching and selecting articles based on the PRISMA flowchart. We found a total of 15,064 articles by searching in PubMed, Web of Science, and Scopus databases. After removing duplicates (*n* = 6537), We took three steps: (1) screening the article titles, (2) reviewing the article abstracts, and (3) reviewing the full text of the articles and extracting the data by the second group. Based on the predetermined criteria, we eliminated 8,408 studies during the one and two-stage process. In the third stage, from 119 articles unrelated studies (*n* = 86), articles with unavailable full text (*n* = 8), review studies (*n* = 4), and book chapters (*n* = 1) were excluded. Finally, 20 articles entered the third stage, i.e., a review of the full text of the articles. The research team extracted required data elements such as publication year, country, journal/conference, purpose, study design, sample size, sample age range, results, tools, challenges/limitations, Relevance to the study and system target. We recorded the extracted data in an Excel (Table 1).

**Table 1:** Summary of included contributions (n=20)

Author (Ref.)	Publication year	Country	Journal	conference	study design	Study aim(s)	Sample size	Sample description	Tool	Results	Challenges and limitation	Relevance to the study			AI (AI) algorithms	System target	
												Diet	AI	Application		anticipation	diagnostic
Lehtinen et al. [21]	1997	Finland	*		Case study	Comparing the performance of SOM and TPFFN in anticipating the possibility of PCOS	Patients: 54 Control group: 29	27 + 33 +		TPFFN accuracy was better than SOM.	Small sample data volume		*		SOM, TPFFN, MLP	*	
Zhang et al. [22]	2010	USA	*		RCT	Construction of classification models for the anticipation of the occurrence of ovulation treatment in women with PCOS	418	Clomiphene citrate: 27.9 + 4.0 The combination of clomiphene citrate and metformin: 28.3 + 4.0		Clomiphene citrate alone is better and superior to the other two methods for treating PCOS.			*		Decision trees	*	
Mehrotra et al. [2]	2012	India		*	Original	described a method that Enables automatic diagnosis of PCOS based on features	Normal: 150 abnormal: 50	Normal: 32.24 ± 2.02 Abnormal: 31.24 ± 2.48		Bayesian classifier gives higher accuracy than logistic regression. Using the probabilistic model helps doctors to screen early patients who are more likely to develop the disease.	Need to improve accuracy by using other classifiers		*		Bayesian Classifier, Multivariate LR		*
Rethinavalli et al. [23]	2016	India	*		Original	Proposing a new combinatorial structure to discover the severity of the disease in people with the disease	31		SQL MATLAB R 2016 a Dataset: Polycystic Ovarian Syndrome Proliferative Phase Endometrial Cell Types	The structure based on fuzzy logic can be used in risk anticipation The severity of the disease was improved. The proposed model performed better than the other created models with an accuracy of 93.64%			*		NFRS, ANN	*	
Cahyono1 et al. [24]	2017	Indonesia		*	Original	Designing and creating a system based on convolutional neural network to classify ultrasound images into two categories, sick and healthy	Patient: 40 Healthy: 14		3D matrix Softmax Loss function Dropout SGD method F1-Measure Micro-average F1-Measure	Automatic classification of images into two categories, sick and healthy, by the designed system It was done well and was very accurate			*		CNN		*
Dewi et al. [25]	2018	Indonesia		*	Original	System design based on machine learning and AI to help Doctors can diagnose the disease more easily through ultrasound images			Gabor Wavelet method	The use of competitive neural network can increase the accuracy of diagnosis in this article The highest accuracy is estimated at 80.84%. According to the results, the number of adopted features has a direct relationship with accuracy			*		Competitive Neural Network		*
Thufailah et al. [26]	2018	Indonesia		*	Original	System design based on the Gibber-Violet method to extract features and Helping to diagnose and classify disease	16–32 features		Gabor Wavelet method	The best accuracy of using the elemental neural network was 78.1%, which was achieved with 32 features. A higher number of data for training the network can increase the accuracy of the network	More data for training affects the time of diagnosis		*		Elman Neural Network, Polynomial SVM, Radial Basis Function SVM, Linear SVM		*
Vikas et al. [27]	2018	India	*		Original	Identify recurring patterns among the symptoms of PCOS patients using a set of frequently used items	119	18–22	PCOS Dataset source: https://github.com/PCOS-Survey/PCOSData Frequent Itemset Mining (FIM) Spss	Using the mentioned algorithm to extract the main widgets Here, the main signs have performed well for anticipation as well as determining relationships between features	The data set used is not enough. In addition, Patients’ concerns about information disclosure	*	*		Apriori algorithm	*	
Denny et al. [28]	2019	India		*	Original	Designing and creating a system based on AI for assistance To diagnose and anticipate PCOS disease	patients: 177 Healthy: 364	18–40	SPSS V 22.0 Principal Component Analysis (PCA) Spyder Python IDE HTML with SQL for designing a proper user interface	Among the algorithms used, Algorithm RF performed best with 89% accuracy. The system designed according to experts can be useful in early disease diagnosis and save time.			*		NB, LR, KNN, CART, RF, SVM	*	*
Thakre et al. [18]	2020	India	*		Original	Design and build system based On AI for help to diagnose and anticipate PCOS disease	30 features		Jupyter Notebook Python	This system helps in the early diagnosis and prediction of PCOS, and the RF algorithm is the most accurate and reliable algorithm with an accuracy of 90.9.			*	*	RF, LR Linear SVM, Radial SVM, KNN, Gaussian Naive Bayes	*	*
Abu Adla et al. [29]	2021	Lebanon		*	Original	Designing a proposed model for automatic diagnosis of PCOS	Patients: 177 Healthy: 364	18–40	“Polycystic Ovary Syndrome”dataset, ML application	The best performance was related to the linear support vector machine, which was 90% accurate with 24.	Despite high accuracy in automatic model recognition Suggestions did not show good performance in recall		*		SFFS, LR, DT, NB, Linear SVM, Polynomial SVM, Radial Basis Function SVM, Linear Discriminant Classifier, Quadratic Discriminant, RF		*
Hassan et al. [30]	2020	India	*		Original	Design and build system based on AI for help to diagnose PCOS and compare the performance of different algorithms	42 variables		R-language R libraries: e1071, CARET, naivebayes, rpart, randomForest, klaR, ggplot2	Among the 5 algorithms used, RF algorithm and support vector machine respectively Accuracy of 96% and 95% performed better.			*		LR, SVM NB, CART, RF		*
Kodipalli et al. [31]	2021	India	*		Original	Designing a model for disease anticipation and related mental disorders based	624	Patients under 25	Questionnaire, K10 tool, matplotlib, Fuzzy TOPSIS	The use of the system is cost-effective. The performance of SVM and fuzzy algorithms was 94.01% and 98.2%, respectively.			*		D-Tree, KNN, SVM, Fuzzy	*	
Song et al. [32]	2022	China	*		Original	This study proposed a model based on Artificial intelligence algorithm, which is a non-invasive method with the help of captured images It was from the eyes to help diagnose PCOS.	721		U-Net network, convolutional block attention module (CBAM), multi-instance (MIL), MLP, Resnet18	A non-invasive method, The accuracy of this method was estimated at 0.978%.	Ambiguities in the images, There is a need to conduct more studies to generalize the results		*		CNNs: V3, Vgg16, and Vgg19		*
Mandal et al. [16]	2021	India		*	Original	Providing an automated diagnostic approach for Detection of follicles in the ovary using ultrasound (US) images during infertility treatment.	19		histogram equalization	This method can automatically detect the follicles Ultrasound images are effective in reducing the workload of doctors.	To determine the exact shape and size of the follicles There are more features that need to be considered.		*		K-means clustering		*
Nilofer et al. [33]	2021	India	*		Original	Presenting a proposed method for automatic division of areas in ultrasound images into areas with follicles and without follicles.			Wiener filter, Takagi–Sugeno–Kang (TSK), fuzzy inference method, Maximum Likelihood (ML), Extreme Learning Adaptive Neuro-inference System (ELANFIS)	The proposed combined model had 99% accuracy in detecting follicles.	Further research is needed to be done by institutions and stakeholders to confirm the model.		*		Fuzzy logicis, Hybrid, Intelligent Water Drop (IWD), KNN, SVM		*
Zhang et al. [34]	2021	China	*		Original	Designing a system based on deep learning for the anticipation of diseases related to genetics including PCOS	Thousans of genetic variants		DisGeNET, GWAS Catalog, GTEx Portal	The current algorithm in the field of predicting the relationship of disease with genetics compared with algorithms Classics such as RF and Support Vector Machine performed better.			*		CNN, GCN	*	
Hosain et al. [35]	2022	Bangladesh		*	Observational study	Development of a system called PCONet To help diagnose pcos through convolutional neural network-based ultrasound images	Dataset 1: 1730 images Dataset 2: 339 images		Image Data Generator, Keras	The present system not only performed well in diagnosing the disease through images, but also performed better with an accuracy of 98.12.			*		CNN, InceptionV3		*
Zigarelli et al. [36]	2022	United States of America	*		Retrospective study	developing self-diagnostic prediction models for PCOS in potential patients and clinical providers	541	20–48	Rotterdam criteria PCA Method	The prediction accuracy was estimated to be 87.5 to 90.1%	The sample was drawn from a specific population in India from several hospitals.		*		K-Means Clustering, CatBoost model		*
Nsugbe et al. [37]	2023	England	*		Original	Designing and creating a decision support system based on AI to diagnose PCOS and determine the stage of the disease	Patients: 177 Healthy: 364		Kaggle website	SVM performed better than other used algorithms.	More samples with more diverse data for presenting the model in the clinical environment is needed		*		DT, LDA, LR, KNN, SVM		*

### Publication analysis

#### Distribution of studies by year

**Fig. 2:**
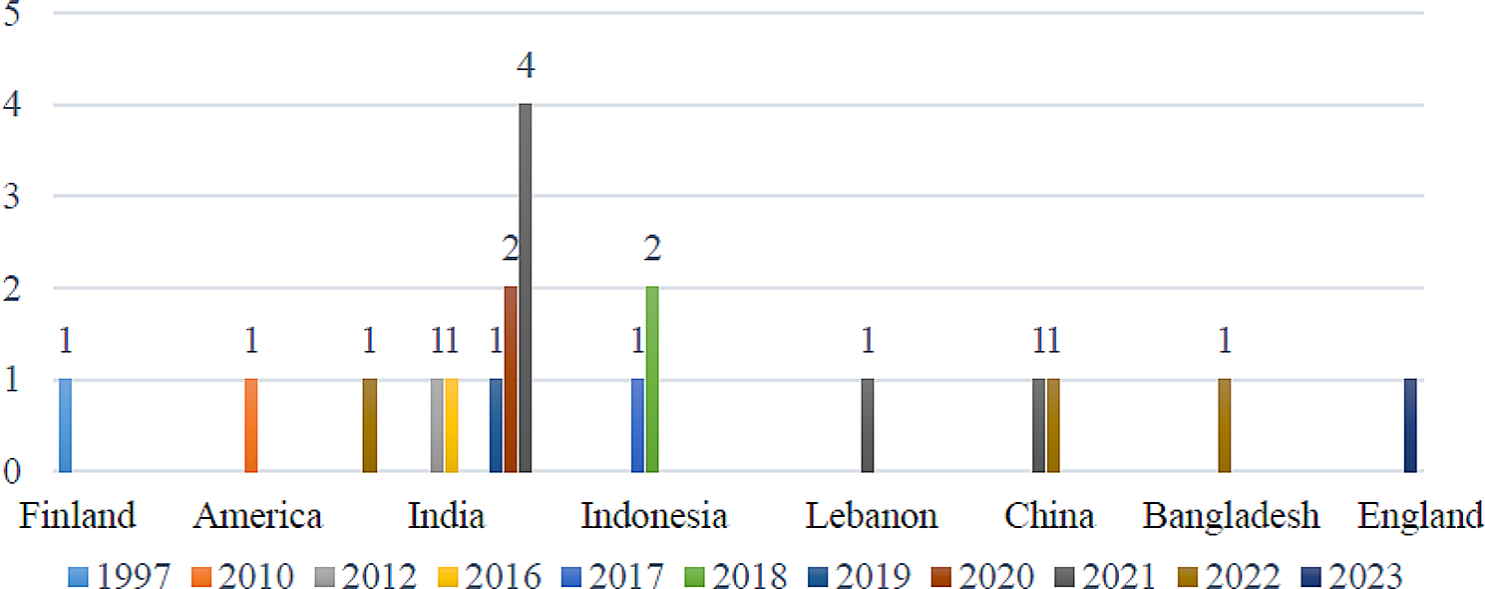
Distribution of studies by country and year

The studies were conducted from 1997 to 2023. Figure 2 shows the results. 2021 (*n* = 5) has the most frequency. The second frequency was related to 2018 and 2022. (*n* = 3).

#### Distribution of studies by country

**Fig. 3:**
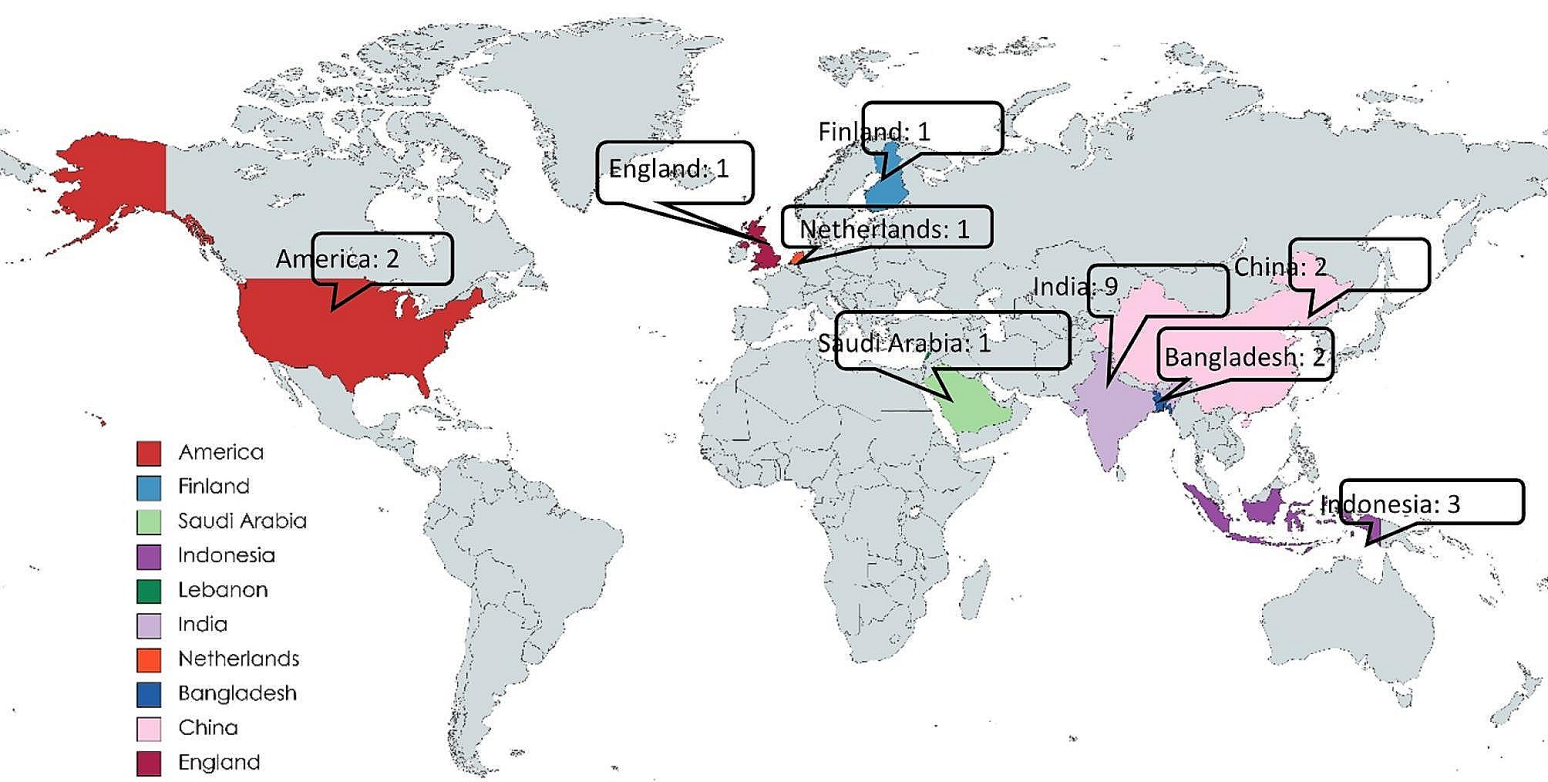
Distribution of studies by country

The reviewed studies were conducted in eight different countries. The frequency of these studies in each country is depicted in Figs. 2 and 3. India had the highest number of studies (*n* = 9), followed by Indonesia (*n* = 3), the United States of America, and China (each with 2), ranking second and third, respectively.

#### Distribution of articles based on journal/conference name, publisher and impact factor

The articles were appeared in 11 different journals and seven conferences. The journal “Frontiers in Endocrinology” had the highest number (*n* = 2), while all the other journals had only published 1 article. All conferences, except the “International Conference on Data and Information Science,” presented a paper on the topic. Tables 2 and 3 display the distribution of articles in this field.

**Table 2:** Distribution of articles based on journal/conference name, publisher and impact factor

Journal/conference name	Cite Score quartile	Publisher	Indexed in (ISI, Scopus, PubMed)	IF	Count of papers
Human Reproduction	Q1	ProQuest	ISI, Scopus, PubMed	6.1	1
International Journal of Circuit Theory and Applications (I J C T A)	Q1	Wiley	ISI, Scopus	2.3	1
Information Systems Design and Intelligent Applications	-	-	-	-	1
Bioscience Biotechnology Research	-	-	-	-	1
International Journal of Computer Applications	Q2	Other	ISI, Scopus	1.1	1
Webology		Other			1
Frontiers in Cell and Developmental Biology	Q2	Other	ISI, Scopus, PubMed	5.5	1
Journal of Medical Internet Research (JMIR)	Q1	Other	ISI, Scopus, PubMed	7.4	1
Healthcare Analytics	-	Other	Scopus		1

**Table 3:** Distribution of articles based on conference name

Conference name	Count of papers
IEEE India Conference (INDICON)	1
International Conference on Information and Communication Technology (ICOICT)	1
International Conference on Data and Information Science	2
IEEE Region 10 International Conference Tencon	1
International Conference on Advances in Biomedical Engineering (ICABME)	1
Proceedings Of International Conference on Frontiers in Computing and Systems	1
International Conference on Engineering and Emerging Technologies (ICEET)	1

### Study specifications

#### Frequency of studies based on AI/application

Based on the survey, most (95%) of the studies focused on models and systems utilizing AI technology [2, 16, 21–37], while only one study resulted in the creation of an AI-based application [18].

**Frequency of studies based on the type of system application**: When it comes to systems and applications designed for various purposes, they can be classified into three types: prediction, diagnosis, and management. Among all the conducted studies, 60% focused on diagnosis [2, 16, 24–26, 29, 30, 32, 33, 35–37], 30% on prediction [21–23, 27, 31, 34], and none on management. Additionally, 10% of studies focused on both diagnosis and prediction [18, 28]. However, none of the studies examined the role of nutrition in managing PCOS.

#### Specifications of performance metrics for model evaluation

Table 4 displays the metrics utilized in the articles. MAE [23], RMSE [23, 33], and RRSE [23] calculate three types of errors in implemented models, so the lowest value is considered for an ideal model. MAE in mathematics is the arithmetic equivalent of absolute errors. This criterion only measures the magnitude of the error and does not give a significant indication of the direction of the error. These three criteria have been used in a study to evaluate the model’s performance [23, 33].

The Kappa statistic is a tool that evaluates the effectiveness of a model’s reliability and ensures an accurate representation of changes in collected data. It can range from − 1 to + 1. Despite being a common statistic, there is no consensus on measures of it in health studies. It has been utilized solely in one study [38].

Most studies commonly used Sensitivity [2, 18, 28–30, 32–37], Specificity [2, 28], and Accuracy [2, 18, 26, 28–30, 32–37] as criteria. These metrics measures by True Positive (TP), True Negative (TN), False Positive (FP), and False Negative (FN). F1 score or 𝐹 − 𝑀𝑒𝑎𝑠𝑢𝑟e as a harmonic average of accuracy and recall. Where there is a balance between accuracy and recall, it is a better metric [2, 18, 26, 28–30, 32–37].

**Table 4:** Performance metrics for model evaluation

Metric	Formula	Range	Desirable
𝑃𝑟𝑒𝑐𝑖𝑠𝑖𝑜n	𝑇𝑃/ 𝑇𝑃 +𝐹𝑃	0-100%	Max
Sensitivity/ Recall	𝑇𝑃 /𝑇𝑃 + 𝐹𝑁	0-100%	Max
Specificity	𝑇𝑁 /𝑇𝑁 + 𝐹𝑃	0-100%	Max
Accuracy	𝑇𝑃 + 𝑇𝑁/𝑇𝑃 + 𝑇𝑁 + 𝐹𝑃 + 𝐹𝑁	0-100%	Max
𝐹 − 𝑀𝑒𝑎𝑠𝑢𝑟e	(2 * Precision * Recall) / (Precision + Recall)	0–1	Max
Positive Predictive Value (PPV)	(sensitivity * prevalence) / [ (sensitivity * prevalence) + ((1 – specificity) * (1 – prevalence))	0-100%	Max
Kappa statistic	2*(𝑇𝑃 *𝑇𝑁- 𝐹𝑁* 𝐹𝑃)/(𝑇𝑃+ 𝐹𝑃)*(𝐹𝑃+ 𝑇𝑁)*(𝑇𝑃+ 𝐹𝑁)*(𝐹𝑁+ 𝑇𝑁)	0–1	Max
Mean Absolute Error (MAE)	(1/n) Σ(i = 1 to n) |y_i – ŷ_i|	LB:0	Min
Root Mean Squared Error (RMSE)	√ Σ(i = 1 to n) (y_i – ŷ_i)^2^/𝑁	LB:0	Min
Root Relative Squared Error (RRSE)	√Σ(i = 1 to n) (ti − ri)^2^ /Σ(i = 1 to n) (ti−ˉt)^2^	0-∞	Min
Area Under the Curve (AUC)		0–1	Max
Precision-Recall Curve (AUPR)		0–1	Max

### Specifications of decision support systems for PCOS prediction

As presented in Table 1, Eight studies [18, 21–23, 27, 28, 31, 34] designed a model to predict disease. These studies used Decision trees [22, 31], Topology-preserving forward network [21], multi-layer perceptron [21], NFRS [23], Artificial neural network [23], Apriori algorithm [27], NB classifier method [28], LR [18, 28], KNN [18, 28, 31], CART [28], RF Classifier [18, 28], Gaussian Naive Bayes [18], Fuzzy [31], CNN [34], GCN techniques [34] and SVM [18, 28, 31].

### Specifications of decision support systems for PCOS diagnosis

Automated detection models based on database: Eight studies [2, 18, 28–30, 32, 36, 37] have implemented automated detection models based on a database to diagnose or screen patients automatically. These studies aimed to diagnose diseases automatically by creating an AI-based model using readily available data or data from those who seek treatment at health centers. The studies all followed a similar methodology. They first collected data from healthy and sick individuals. After that, they performed pre-processing to identify parameters and characteristics. They designed the model using selected techniques and evaluated its performance using model evaluation metrics. The studies utilized various methods such as LR [2, 28–30], Bayesian classifier [2], DT [29, 37], SVM [18, 28–30, 37], CNN [32], KNN [18, 28], quadratic discriminant classifier [18, 29], RF [18, 28–30], CART [28, 30], Gaussian naive Bayes [18], and K-means clustering [36] to develop the automated diagnosis model.

Classification models based on images: Four studies [24–26, 35] have developed a diagnostic model for this disease using ultrasound image classification. Ultrasound images were used to train and test the model., and different methods such as CNN [24–26], and SVM [26] were used in these studies.

Follicle segmentation models: Two studies [16, 33] created a model to segment follicles in ultrasound images for automatic disease diagnosis. The model specifically diagnoses through follicle segmentation, reducing the time needed for follicle counting. The process began with the publication of images, followed by image processing to segment the follicles. After extracting features, classifiers were used to design the model. Techniques used during the pre-processing stage included histogram equalization, contrast enhancement, and the Wiener filter for noise reduction of the images. The segmentation stage utilized the Fuzzy logicis, Hybrid Intelligent, Water Drop (IWD), KNN, SVM [33] and K-means clustering [16].

### Limitations and challenges mentioned in the studies

As presented in Tables 1 and 10 studies pointed out the limitations and refinements. Accordingly, the small volume of sample size and features in 4 studies, the need to conduct more studies in 3 studies, the increase in the time of automatic diagnosis by the system by using more data in one study [26], the unwillingness of patients to disclose reports and clinical data in one [19] and the need to improve accuracy using other classifiers is mentioned in one study [2].

### Quality assessment of included studies

The quality assessment of the included studies is detailed in the Supplementary file (Table S4), with 17 studies rated as high quality and three as moderate qualities.

## Discussion

The purpose of this study was to conduct a thorough review of recommendation systems for women with PCOS. Specifically, we focused on models or applications that utilized artificial intelligence. We collected information from various sources such as publication year, country, journal or conference, sample size, age of participants, limitations and challenges, and results. During systematic review, we found five studies that shared a similar approach [2, 11, 12, 14, 17, 19]. . We found reasons for using mobile or AI-based recommender systems in PCOS disease management. We will now delve into the study’s findings and other studies.

Abhari et al. investigated nutritional recommendation systems without considering a specific disease. In this study, we reviewed the proposed recommender systems for polycystic ovary syndrome with its various applications [14].

Based on the study results, obesity in people with PCOS, with the escalation of symptoms, increases the cost of treatment and reduces it, especially in infertility. The expenditure of lifestyle modification with the help of health and weight loss is lower than drug therapy. Modifying lifestyle and nutrition using mobile phones is considered a low-cost intervention with a lower percentage of invasion [20]. The results of a 2018 study by Jacqueline A. Boyle and colleagues in Australia showed that a quality disease management application met the needs of patients; however, none of the applications reviewed had quality [12].

As mentioned, early diagnosis of the disease in the early stages is associated with risk reduction of disease consequences. Therefore, we may need recommender systems to reduce risk reduction and time of the diagnosis and increase accuracy [32]. One study by Naila Jan and colleagues in 2023 investigated AI techniques for PCOS diagnosis. The results of this study show that early diagnosis of this disease is difficult despite different symptoms in people, so automatic detection systems can be used as an accurate solution in this field [17]. As with our results, the existence of a limited amount of data is considered as one of the obstacles to the implementation of this type of study [17, 27].

Two studies conducted by Naila Jan and colleagues in India in 2022 reported a PCOS rate of 3.7 to 22.5%, which was higher in urban than rural women [11, 17]. Based on the review, 40.9% of studies have been conducted in India, which justifies the high rate of PCOS. Also, unhealthy lifestyles, including unhealthy eating, can be a reason for most urban women to do this.

Among the models designed to predict PCOS, the best accuracy belongs to a fuzzy logic-based model with an accuracy of 98.2% [31]. In the field of PCOS diagnosis, a hybrid model based on ANN, CNN and InceptionV3 has the best performance among the designed models with an accuracy of 98.12 [35]. RF and SVM were two widely used algorithms with acceptable performance, but the performance of the CNN-based model with 97% accuracy is better than these two algorithms [32]. Based on the study by Naila Jan and colleagues, the best performance belonged to a CNN-based system with an average of 76.36% and a micro-average f1-score of 100%. KNN, ANN, and Fuzzy logic with an accuracy of 97%, 97.5%, and 97.30 were the best classification techniques among the reviewed articles [11, 17].

Another study in 2018 by Jue Xie and colleagues in Australia aimed to investigate the AskPCOS application and the steps involved in creating it. The results showed that the mentioned program is one of the best evidence-based user programs to manage PCOS disease. Due to the support of 5 common languages of the world, it can eliminate the inequality of lack of access to information in developing countries. According to the evaluation, 80% of people were satisfied. The application’s usefulness was 70%. Jacqueline A. Boyle’s study showed that evidence-based application programs positively affect the patient’s awareness [19].

### Study limitations

The study has several limitations. (1) only two studies addressed the aspect of concomitant health management in PCOS. (2) there is a lack of access to several paid articles that can provide better results by reviewing them. 3, the case was a similar study in other developing countries such as Iran, which may cause problems in the results of this study in this country. Therefore, we recommend carrying out more specialized studies about recommender systems. We should use AI to modify lifestyles to nutritional patterns, such as providing a dedicated budget, creating motivation, and creating a culture among researchers.

## Conclusion

We conducted a systematic review to explore the use of AI and companion health systems in managing polycystic ovary syndrome, with a focus on nutrition. Although AI has primarily been used for disease diagnosis, the positive impact of AI and companion health systems in providing nutrition-based treatment solutions is significant. Therefore, we recommend that countries, particularly those with a high number of affected individuals, prioritize policies that encourage further studies to evaluate the effectiveness of recommendation systems on the nutrition of people with polycystic ovary syndrome. We can work on the quality of life for individuals affected by this condition.

### Electronic supplementary material

Below is the link to the electronic supplementary material.

Supplementary Material 1

## Data Availability

The datasets generated and/or analysed during the current study are available in the PubMed, Scopus, and Web of Science databases.

## Abbreviations

AI: Artificial Intelligence
PCOS: Polycystic ovary syndrome
SOM: Self-Organizing Map
TPFN: Topology-Preserving Forward Network
MLP: Multi-Layer Perceptron
NFRS: Neural Fuzzy Rough Set Evaluation
ANN: Artificial Neural Network
KNN: K-Nearest Neighbor
CART: Classification and Regression Trees
SVM: Support Vector Machine
LR: Logistic Regression
RF: Random Forest
SFFS: Sequential Forward Floating Selection
DT: Decision Tree
RCT: Clinical Randomized Trial
NB: Naïve Bayes
GCN: Graph convolutional network
CNN: Convolutional Neural Networks
LDR: Linear Decision Analysis
