# Supplementary material for: Nutritional management recommendation systems in polycystic ovary syndrome: a systematic review
Source: BMC Womens Health. 2024 Apr 12;24:234. doi: 10.1186/s12905-024-03074-3 (PMC11015675; doi:10.1186/s12905-024-03074-3)
Supplement: Supplementary file 1 — Supplementary Material 1 [file 12905_2024_3074_MOESM1_ESM.docx]

**Supplementary file**

**Table S1:** Search strategy in PubMed, Date: 6/6/2023

| **Number** | **Search Strategy** | **Results** |
| --- | --- | --- |
| 1 | "Polycystic Ovary Syndrome"[MeSH Terms] OR "polycystic ovarian syndrome*"[Title/Abstract] OR "ovary syndrome*"[Title/Abstract] OR "polycystic ovary*"[Title/Abstract] OR "stein leventhal syndrome*"[Title/Abstract] OR "stein leventhal syndrome*"[Title/Abstract] OR "stein leventhal*"[Title/Abstract] OR "sclerocystic ovarian degeneration*"[Title/Abstract] OR "ovarian degeneration*"[Title/Abstract] OR "sclerocystic ovary syndrome*"[Title/Abstract] OR "sclerocystic ovar*"[Title/Abstract] OR "ovarian syndrome*"[Title/Abstract] OR "sclerocystic ovarian*"[Title/Abstract] | 23,595 |
| 2 | "Mobile Applications"[MeSH Terms] OR "Decision Support Systems, Clinical"[MeSH Terms] OR "AI"[MeSH Terms] OR "Recommended Dietary Allowances"[MeSH Terms] OR "mobile application*"[Title/Abstract] OR "mobile app*"[Title/Abstract] OR "portable software app*"[Title/Abstract] OR "software app*"[Title/Abstract] OR "application*"[Title/Abstract] OR "software application*"[Title/Abstract] OR "smartphone app*"[Title/Abstract] OR "portable electronic app*"[Title/Abstract] OR "electronic app*"[Title/Abstract] OR "portable electronic application*"[Title/Abstract] OR "electronic application*"[Title/Abstract] OR "clinical decision support system*"[Title/Abstract] OR "clinical decision support*"[Title/Abstract] OR "information system*"[Title/Abstract] OR "software*"[Title/Abstract] OR "software tool*"[Title/Abstract] OR "computer applications software*"[Title/Abstract] OR "computer software application*"[Title/Abstract] OR "computer program*"[Title/Abstract] OR "computer software*"[Title/Abstract] OR "AI*"[Title/Abstract] OR "intelligence*"[Title/Abstract] OR "computational intelligence*"[Title/Abstract] OR "machine intelligence*"[Title/Abstract] OR "computer reasoning*"[Title/Abstract] OR "reasoning*"[Title/Abstract] OR "computer vision system*"[Title/Abstract] OR "computer vision*"[Title/Abstract] OR "system*"[Title/Abstract] OR "vision system*"[Title/Abstract] OR "knowledge acquisition*"[Title/Abstract] OR "knowledge representation*"[Title/Abstract] OR "recommended dietar*"[Title/Abstract] OR "recommended dietary allowance*"[Title/Abstract] OR "dietary allowance*"[Title/Abstract] OR "reference daily intake*"[Title/Abstract] OR "daily intake*"[Title/Abstract] OR "reference dail*"[Title/Abstract] OR "recommended daily intake*"[Title/Abstract] OR "recommended dail*"[Title/Abstract] OR "daily recommended intake*"[Title/Abstract] OR "daily recommend*"[Title/Abstract] OR "recommended intake*"[Title/Abstract] OR "recommended daily allowance*"[Title/Abstract] OR "daily allowance*"[Title/Abstract] OR "allowance*"[Title/Abstract] OR "daily recommended allowance*"[Title/Abstract] OR "recommended allowance*"[Title/Abstract] OR "dietary reference intake*"[Title/Abstract] OR "intake*"[Title/Abstract] OR "reference intake*"[Title/Abstract] | 6,361,768 |
| 3 | #1 AND #2 | 3,542 |

**Table S2:** Search strategy in Scopus, 6/6/2023

| **Number** | **Search Strategy** | **Results** |
| --- | --- | --- |
| 1 | TITLE-ABS-KEY("polycystic ovarian syndrome*" OR "ovary syndrome*" OR "polycystic ovary*" OR "stein leventhal syndrome*" OR "stein leventhal syndrome*" OR "stein leventhal*" OR "sclerocystic ovarian degeneration*" OR "ovarian degeneration*" OR "sclerocystic ovary syndrome*" OR "sclerocystic ovar*" OR "ovarian syndrome*" OR "sclerocystic ovarian*") | 28,697 |
| 2 | TITLE-ABS-KEY("mobile application*" OR "mobile app*" OR "portable software app*" OR "software app*" OR "application*" OR "software application*" OR "smartphone app*" OR "portable electronic app*" OR "electronic app*" OR "portable electronic application*" OR "electronic application*" OR "clinical decision support system*" OR "clinical decision support*" OR "information system*" OR "software*" OR "software tool*" OR "computer applications software*" OR "computer software application*" OR "computer program*" OR "computer software*" OR "AI*" OR "intelligence*" OR "computational intelligence*" OR "machine intelligence*" OR "computer reasoning*" OR "reasoning*" OR "computer vision system*" OR "computer vision*" OR "system*" OR "vision system*" OR "knowledge acquisition*" OR "knowledge representation*" OR "recommended dietar*" OR "recommended dietary allowance*" OR "dietary allowance*" OR "reference daily intake*" OR "daily intake*" OR "reference dail*" OR "recommended daily intake*" OR "recommended dail*" OR "daily recommended intake*" OR "daily recommend*" OR "recommended intake*" OR "recommended daily allowance*" OR "daily allowance*" OR "allowance*" OR "daily recommended allowance*" OR "recommended allowance*" OR "dietary reference intake*" OR "intake*" OR "reference intake*") | 27,442,116 |
| 3 | #1 AND #2 | 7,208 |

**Table S3:** Search strategy in Web of Science, Date:6/6/2023

| **Number** | **Search Strategy** | **Results** |
| --- | --- | --- |
| 1 | TS=("polycystic ovarian syndrome*" OR "ovary syndrome*" OR "polycystic ovary*" OR "stein leventhal syndrome*" OR "stein leventhal syndrome*" OR "stein leventhal*" OR "sclerocystic ovarian degeneration*" OR "ovarian degeneration*" OR "sclerocystic ovary syndrome*" OR "sclerocystic ovar*" OR "ovarian syndrome*" OR "sclerocystic ovarian*") | 25,096 |
| 2 | TS=("mobile application*" OR "mobile app*" OR "portable software app*" OR "software app*" OR "application*" OR "software application*" OR "smartphone app*" OR "portable electronic app*" OR "electronic app*" OR "portable electronic application*" OR "electronic application*" OR "clinical decision support system*" OR "clinical decision support*" OR "information system*" OR "software*" OR "software tool*" OR "computer applications software*" OR "computer software application*" OR "computer program*" OR "computer software*" OR "AI*" OR "intelligence*" OR "computational intelligence*" OR "machine intelligence*" OR "computer reasoning*" OR "reasoning*" OR "computer vision system*" OR "computer vision*" OR "system*" OR "vision system*" OR "knowledge acquisition*" OR "knowledge representation*" OR "recommended dietar*" OR "recommended dietary allowance*" OR "dietary allowance*" OR "reference daily intake*" OR "daily intake*" OR "reference dail*" OR "recommended daily intake*" OR "recommended dail*" OR "daily recommended intake*" OR "daily recommend*" OR "recommended intake*" OR "recommended daily allowance*" OR "daily allowance*" OR "allowance*" OR "daily recommended allowance*" OR "recommended allowance*" OR "dietary reference intake*" OR "intake*" OR "reference intake*") | 13,458,924 |
| 3 | #1 AND #2 | 4,314 |

**Table S4:** Quality assessment of included studies

| **Author (Ref.)** | **Q1** | **Q2** | **Q3** | **Q4** | **Q5** | **Q6** | **Q7** | **Q8** | **Q9** | **Q10** | **Q11** | **Raw score and %** | **Quality** |
| --- | --- | --- | --- | --- | --- | --- | --- | --- | --- | --- | --- | --- | --- |
| Lehtinen et al. (15) | 1 | 2 | 2 | 1 | 1 | 2 | 1 | 2 | 1 | 0 | 1 | 14; 63.6% | Moderate |
| Zhang et al. (24) | 1 | 1 | 2 | 2 | 1 | 2 | 1 | 2 | 1 | 0 | 2 | 15; 68.1% | Moderate |
| Mehrotra et al. (3) | 2 | 2 | 2 | 2 | 1 | 2 | 2 | 2 | 1 | 0 | 2 | 18; 81.8% | High |
| Rethinavalli et al. (25) | 1 | 1 | 2 | 2 | 1 | 2 | 2 | 1 | 2 | 0 | 2 | 16; 72.7% | High |
| Cahyono1 et al. (26) | 1 | 1 | 1 | 2 | 1 | 2 | 2 | 1 | 1 | 0 | 2 | 14; 63.6% | Moderate |
| Dewi et al. (27) | 1 | 2 | 2 | 2 | 1 | 2 | 2 | 2 | 1 | 0 | 2 | 17; 77.2% | High |
| Thufailah et al. (28) | 1 | 2 | 2 | 2 | 1 | 2 | 2 | 2 | 1 | 0 | 2 | 17; 77.2% | High |
| Vikas et al. (20) | 1 | 2 | 2 | 2 | 1 | 2 | 2 | 2 | 1 | 2 | 2 | 19; 86.3% | High |
| Denny et al. (29) | 1 | 1 | 2 | 2 | 2 | 2 | 2 | 2 | 2 | 0 | 2 | 18; 81.8% | High |
| Thakre et al. (12) | 2 | 2 | 2 | 2 | 2 | 2 | 2 | 2 | 2 | 0 | 2 | 20; 90.9% | High |
| Hassan et al. (30) | 2 | 2 | 2 | 2 | 2 | 2 | 2 | 2 | 2 | 0 | 2 | 20; 90.9% | High |
| Kodipalli et al. (21) | 1 | 2 | 2 | 2 | 2 | 2 | 2 | 2 | 2 | 2 | 2 | 21; 95.4% | High |
| Mandal et al. (11) | 2 | 2 | 1 | 2 | 1 | 2 | 2 | 2 | 2 | 0 | 2 | 18; 81.8% | High |
| Nilofer et al. (31) | 2 | 2 | 2 | 2 | 1 | 2 | 2 | 2 | 2 | 0 | 2 | 19; 86.3% | High |
| Zhang et al. (32) | 2 | 2 | 1 | 2 | 1 | 2 | 2 | 2 | 2 | 0 | 2 | 18; 81.8% | High |
| Song et al. (23) | 2 | 2 | 2 | 2 | 2 | 2 | 2 | 2 | 2 | 2 | 2 | 22; 100% | High |
| Hosain et al. (22) | 2 | 2 | 2 | 2 | 2 | 2 | 2 | 2 | 2 | 0 | 2 | 20; 90.9% | High |
| Zigarelli et al. (33) | 2 | 2 | 2 | 2 | 2 | 2 | 2 | 2 | 2 | 2 | 2 | 22; 100% | High |
| Nsugbe et al. (34) | 2 | 2 | 2 | 2 | 2 | 2 | 2 | 2 | 2 | 2 | 2 | 22; 100% | High |
